# Architecture of a Framework for Providing Information Services for Public Transport

**DOI:** 10.3390/s120505290

**Published:** 2012-04-26

**Authors:** Carmelo R. García, Ricardo Pérez, Álvaro Lorenzo, Alexis Quesada-Arencibia, Francisco Alayón, Gabino Padrón

**Affiliations:** Institute for Cybernetics, University of Las Palmas de Gran Canaria, Campus de Tafira, Las Palmas de Gran Canaria, 35017 Las Palmas, Spain; E-Mails: rperez@dis.ulpgc.es (R.P.); gsamsagaz@gmail.com (A.L.); aquesada@dis.ulpgc.es (A.Q.-A.); falayon@dis.ulpgc.es (F.A.); gpadron@dis.ulpgc.es (G.P.)

**Keywords:** ubiquitous computing frameworks, service-orientation, intelligent transport systems

## Abstract

This paper presents OnRoute, a framework for developing and running ubiquitous software that provides information services to passengers of public transportation, including payment systems and on-route guidance services. To achieve a high level of interoperability, accessibility and context awareness, OnRoute uses the ubiquitous computing paradigm. To guarantee the quality of the software produced, the reliable software principles used in critical contexts, such as automotive systems, are also considered by the framework. The main components of its architecture (run-time, system services, software components and development discipline) and how they are deployed in the transportation network (stations and vehicles) are described in this paper. Finally, to illustrate the use of OnRoute, the development of a guidance service for travellers is explained.

## 1. Introduction

This paper falls within the context of intelligent public transport systems, particularly the technologies for developing and running software systems that provide information services to users of public transportation. One way to improve transport systems is to provide passengers with useful information services. Often, these services require complex software systems that need a high degree of interoperability because they have to operate in a wide range of technological contexts and serve a massive number of users, as is the case with large public transport systems. Therefore, it is advisable to have platforms and environments that facilitate the production and execution of reliable software for these services.

In this paper, we describe a framework, known as OnRoute, for producing and running software that provides information services for public transport passengers. The environment provides a set of operating principles and components from which services, such as on-route guided assistance, information at stops and payment systems, can be developed. For the purposes of OnRoute, data relating to the position of the vehicle and planning, as well as the local communications system, are the components demanded of the on-board infrastructure. OnRoute services are accessible through users' mobile devices, e.g., mobile telephones and Smart Phones, using Bluetooth communication infrastructure that is installed at important points of the transport network, such as stations, stops and vehicles. This framework has been used to develop three examples of information services. The first example is an on-route guidance assistant that provides the passengers of a public transport bus with information pertaining to points of interest along the route, the next stop and the estimated time of arrival at the next stop or the final stop. The second example is a payment system that allows travellers to pay the bus fare. The third example is an inspection system that permits the transport company staff to access vehicle information on route to validate the number of passengers on-board and the different operations conducted during travel. In all cases, users have to use their mobile devices (mobile or smart phones) to access the services provided by the public transport vehicle.

OnRoute uses the paradigms of ubiquitous computing [1] to produce and provide support to software that offers public transport information services aimed at users who interact in contexts of mobility, more specifically, passengers, drivers and operations control staff. Services of this type require the resources used by the transport network infrastructure to automate their production activities. We first highlight the on-board systems that encompass all of the devices installed on board the buses to control the services that the vehicle offers, support the payment systems and handle the relevant events that occur during the operations conducted in the vehicle. For the purposes of OnRoute, data relating to the position of the vehicle and planning along with the local communications system are the components demanded of the on-board infrastructure. OnRoute also uses the local communications infrastructure installed at important points of the transport network, such as stations and stops. Specifically, the communication infrastructures used by OnRoute are Bluetooth for communication with user mobile terminals and Wifi for communication with the transport infrastructure. For example, a vehicle must always adhere to the timetable, reaching each stop at the correct time. If the vehicle does not, the on-board system must detect this planning breach and report it in real time to take the necessary measures to minimise the negative impact on the customer. Such measures would include communication with the OnRoute information services using the vehicle's Wifi infrastructure.

This paper is structured as follows: Section 2 describes a set of works related to the topics of this paper; the technology used is presented in the third section. The fourth section is dedicated to explaining the requirements of the OnRoute framework. The OnRoute architecture is described in Section 5, which explains the main elements of the architecture from two points of view: operation principles and software development. Section 6 offers an illustration of how to use the OnRoute framework to implement an on-route guidance assistant. Finally, the main conclusions and future work are presented in the last section.

## 2. Related Works

The potential offered by intelligent transport systems for improving public transport is described in many of the papers listed in the bibliography. For example, Giannopoulus [2] analyses how information and communication technology can be used to develop systems that offer passengers information, adapting it to different operating environments and to different kinds of passengers.

The development of ubiquitous or pervasive systems in the context of transport falls into three categories: contributions that describe frameworks, works that propose an alternative model based on middleware, and works that propose models for the development of pervasive applications that require codes to implement planning and optimisation algorithms. In the first group, Meier [3] proposes the iTransIT framework to integrate transport systems that model spatial information and to implement the spatial application programming interface for pervasive information services, such as smart traveller information services. In the case of middleware, Gluli [4] describes a service-oriented middleware solution and its implementation, in a demonstration vehicle, assuming the non-functional requirements of security, privacy, usability, and reliability (SPUR). In the third group, Ossowski *et al.* [5] introduce a social multi-agent decision support system architecture and provide design guidelines to construct an agent-based decision support system. To illustrate the method, it is applied to two classic transport problems: road traffic management and bus fleet management. Harrington and Cahill [6] propose a model-driven approach to developing pervasive applications. In particular, these authors address how to generate code to implement planning and optimisation algorithms in pervasive environments. These authors present an empirical evaluation of the model impact on the development of a pervasive application in the domain of intelligent transportation systems, such as the optimisation of traffic light settings in an urban traffic control system to minimise the waiting time for vehicles.

In the context of ubiquitous software, Lopez de Ipiña *et al.* [7] have devised a framework for the development and deployment of smart objects and the transformation of a user's mobile device into a universal remote controller. Barretto [8] proposes a framework for developing and running sensor-based, context-aware agents that are based on re-usable components, while Römer [9] proposes a framework based on Jini and Web Services. García-Herranz [10] proposes a programming system that allows the end users to control and programme their environments through a uniform, application–independent method.

## 3. Domain Model of OnRoute: System Requirements

The system that we describe in this paper is a framework to programme and execute mobile applications that provide information services in the public transport context. These information services have a high degree of accessibility by the users (travellers, transport company staff and regulatory agency staff). The services provided by applications that have been developed using OnRoute are accessible through general-purpose mobile terminals that do not necessarily perform well. Therefore, access to the services must be through local wireless communication networks that are available to these kinds of devices. Moreover, the mobile device applications developed using OnRoute are Java applications built using the Java Platform Micro Editions (the applications developed by the OnRoute framework will run in the public transport domain), the proposed architecture meets the following requirements:

Heterogeneity of mobile devices. Services should be available to a variety of mobile devices.
Scalability. The system allows new elements to be added to the infrastructure that permit newly developed information services to be added or make them accessible to a greater number of users.
Spontaneous interaction. The system allows for the spontaneous interaction with users that are already using other system services; this number of users is potentially massive.

These OnRoute requirements can be structured into two groups. The first group is related to the general requirements of the ubiquitous systems, and the second is related to the requirements of the transport information systems.

In terms of the requirements of the ubiquitous systems, the software produced using OnRoute can be characterised by its capacity to integrate its surrounding physical and technological environment. Consequently, it can operate autonomously and spontaneously in different environments. To attain these functionalities, OnRoute accepts the principles that characterise pervasive system software [11]. The boundary principle establishes that the distinction between environments in pervasive frameworks must be made by boundaries that mark differences in content, and these boundaries do not need to limit the interoperability of the systems. The principle of volatility establishes that pervasive systems must accept that the number of users, devices and applications that intervene in a pervasive environment is unpredictable. For this reason, a set of invariable operating principles that govern the running of the system must be specified. Because of these characteristics, the OnRoute architecture is deployed in two areas. The first area is the infrastructure that the public transport system provides. This includes a basic set of components, comprising all of the elements that allow user applications to access transport-related information. The second area is user devices, comprising all of the components that have the capacity to integrate into the different environments and that facilitate access to the information produced by the OnRoute information services.

Following Hervas [12], ontology provides the following benefits: it enables the system to be interoperable, it reduces the difficulties related to technological diversity, and it facilitates communication between humans and computing systems. To facilitate the identification of specific requirements related to the transport information system, the main ontological elements of OnRoute are presented below:

Line. This is the route followed by the vehicles. During the route, the information services are provided to the passengers.
Vehicle. This is the element used to transport passengers. When a vehicle is in service, it follows a route. During the route, information services are provided to the passengers.
Service. This is an abstract entity that models transport data. It offers useful information services to the passengers, such as a payment system or an on-route guidance assistant.
Programmer. This is the person who develops a service and defines the data and the functionalities contained in the service.
Mobile terminal. A device used by the transport client to access the information services.
OnRoute. The active entity, executed in vehicles, that provides information services.
Bluetooth interface. The interface used by mobile terminals to communicate with OnRoute and *vice versa*.

**Figure 1.**
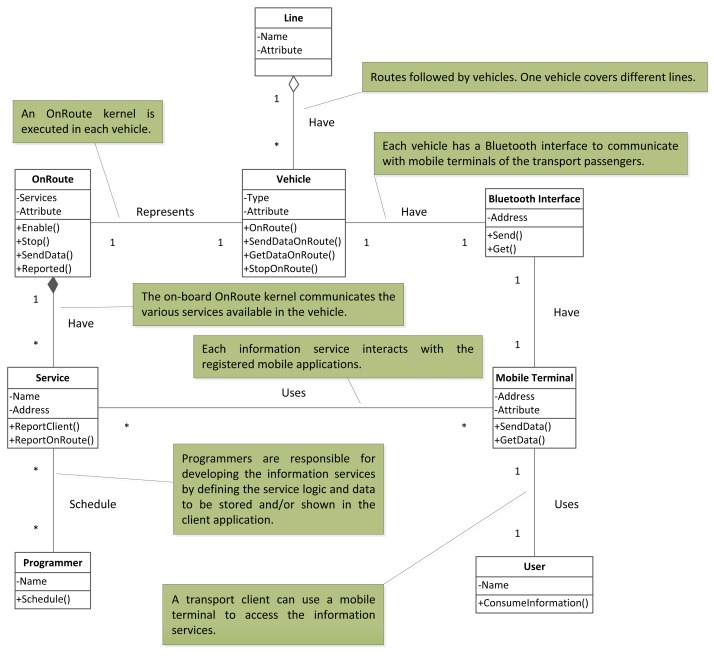
Relations between main ontological concepts.

The relationships between these main concepts of the OnRoute framework are shown in Figure 1.

## 4. OnRoute Architecture

**Figure 2.**
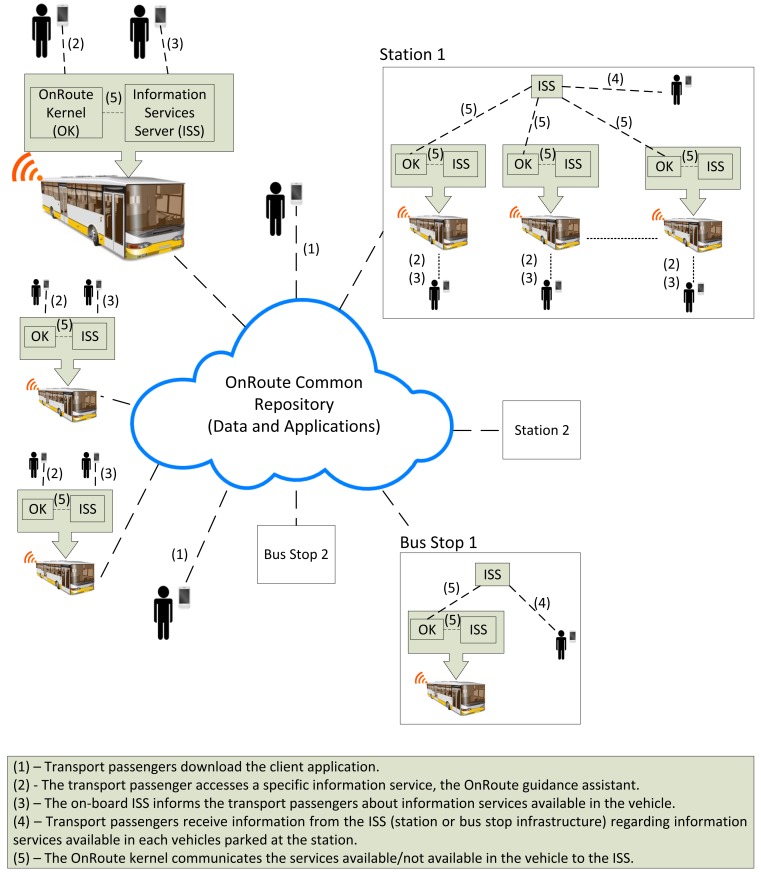
General vision of the OnRoute information services.

OnRoute provides a framework for developing and running software that offers information services to public transport passengers using the existing infrastructures available on board vehicles at stops and in stations. Passengers use their computing and communications devices (mobile telephones and Smart Phones) to access the information generated using OnRoute (see Figure 2). We will have a set of OnRoute services with varying availability and a set of OnRoute client applications that will vary in number. If a passenger is in a station that is busy in terms of both passengers and vehicles, the number can be massive. This situation poses the greatest difficulty for meeting all of the operating requirements and principles described in this section. The reason for this is that a Bluetooth service has to be defined in each OnRoute server, *i.e.*, on each vehicle. The Bluetooth service reports which services are defined in its memory. The OnRoute client applications have to discover these services. Because of the limitations of Bluetooth technology and of the actions of the OnRoute servers, declaring services available and the OnRoute client applications discovering said services must be performed properly. This process is one of the major challenges solved by the OnRoute architecture.

**Figure 3.**
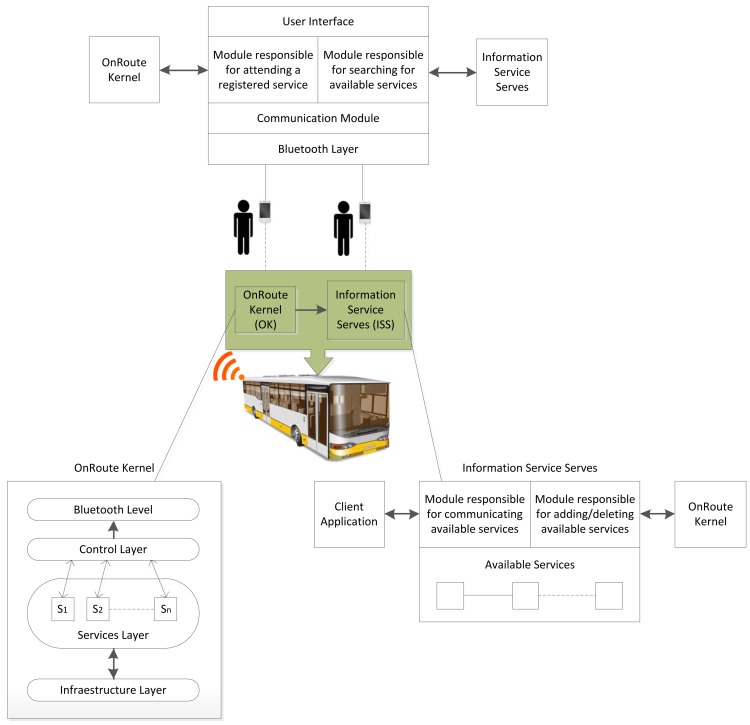
OnRoute general architecture.

The elements of the OnRoute architecture span three domains: the off-board enterprise infrastructure, the nomadic elements (device or service) and the embedded elements (in-vehicle) (see Figure 3). With the OnRoute architecture, when a vehicle reaches a station, a component of the architecture, known as an Information Services Server (ISS), is informed of its availability and its connection address. When the passenger's OnRoute client application seeks available services, it contacts the ISS. The ISS sends the client all of the active services in its database and their connection addresses. The OnRoute client application connects to the OnRoute server in which it is most interested. The OnRoute architecture attains a high degree of service scalability because there is no need for a different service identifier for each OnRoute platform run; they all use the same identifier and report their connection address to the ISS. The time required to search for the services is also reduced because the mobile phones only have to search for a single engine, the ISS. They then connect directly to the line in which they are interested. This arrangement has proved to be fast because the search for devices is slow in devices such as mobile telephones. The more devices there are nearby, the more pronounced is the limitation. The explanation of the Information Services Server, the OnRoute kernel, and the client application will be given below. This description will be composed of two perspectives, that of the operation principles and that of the software development, presenting the main classes, methods and data structures.

### 4.1. The Information Services Server (ISS)

This entity regulates communication between the OnRoute information services that are run in each vehicle and the client applications. This component of the OnRoute architecture must be run in the central mass transport stations. This approach provides a centralised point of access for the client applications to find what services are available in the station at the time and offers the vehicles a simple way to disseminate their active services. The ISS will also be installed in each of the fleet vehicles to allow a passenger who boards at some point other than the central terminus access to the OnRoute services. The on-board ISS will activate when the vehicle leaves a station, and it will deactivate when it reaches another station, thus using the vehicle location system.

**Figure 4.**
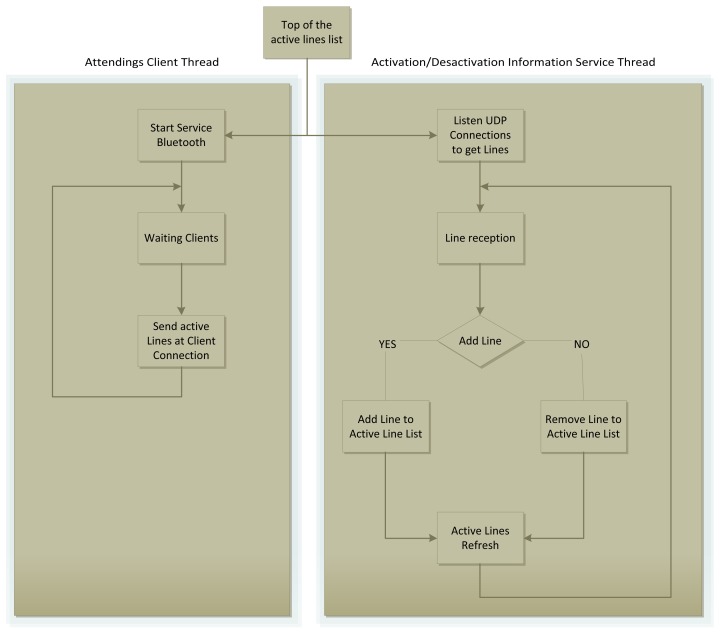
ISS execution flow.

From the implementation point of view, the most important data structure of the ISS is the list of active information services. This list is the first thing that is created. Once the list has been created, running the server consists of two concurrent threads: one to deal with the arrival of client applications that need to know which information services are active in the station and the other to listen to the incoming connections from the vehicles to activate or deactivate the information services (see Figure 4).

One issue that should be borne in mind with regard to the ISS concerns the Bluetooth technology: the maximum range of a Bluetooth device is 10 metres (for Class 2 Bluetooth devices). The ISS will normally be located in central transport terminals. In most cases, one server will cover an area with a diameter of twenty metres. To ensure service coverage throughout the area, the OnRoute architecture makes a replica of the ISS. This solution is valid because of the way that Bluetooth searches for services. First, a search for devices is performed. Found devices are interrogated in search of the desired service. Because all of the servers will have all of the information, if the client application is within the radius of at least one ISS, it will be able to obtain the necessary data.

### 4.2. OnRoute Kernel

This kernel is the main component of the architecture. Therefore, the system has been named after it. The kernel's objectives are clear: (1) to provide passengers with services during their journeys and (2) to provide a development environment to construct services without concern for details, such as data transmission. We can clearly distinguish four layers (see Figure 3). The Infrastructure Layer (IL) provides communications operations with the on-board systems in the mass transport vehicles. This layer makes it possible to obtain data, such as the time remaining until the next stop is reached or the exact geographic location. It can be used by the superior layers, especially by the Services Layer. The Services Layer (SL) is where the services available to the passengers during the journey are located. The Control Layer (CL) manages the flow of information between the SL and the client applications (Bluetooth), together with the synchronisation with the ISS. Finally, the Bluetooth Layer (BL) is responsible for communicating with the mobile user devices. This layer can be generalised to a data exchange layer with the client applications. In this layer, there are communication services for each of the services of the SL.

#### 4.2.1. The Services Layer (SL)

Because of the SL, a programmer need only focus on the problem being addressed and can ignore the infrastructure, such as the communications. To this end, operating principles are defined together with a set of methods that follow these principles. All OnRoute information services consist of a programme that follows the run order below:

**Step 1:** Data initiation. Before anything else, the variables necessary for running the service must be initiated. Certain data will come from outside, while others will be calculated when the service is initiated. This will partially depend on which programmer is developing the OnRoute information service.
**Step 2:** Service publication. Any information service in this layer informs the higher layers that it is starting to operate. Because it is illogical to have services that do not communicate with each other, this step runs constantly, independent of the programmer's intentions.
**Step 3:** Running the service logic. For all intents and purposes, this is the programme itself. Code is encapsulated and run here. Methods of the CL will be used to establish a dialogue between the service and the remote applications in the mobile devices.

**Figure 5.**
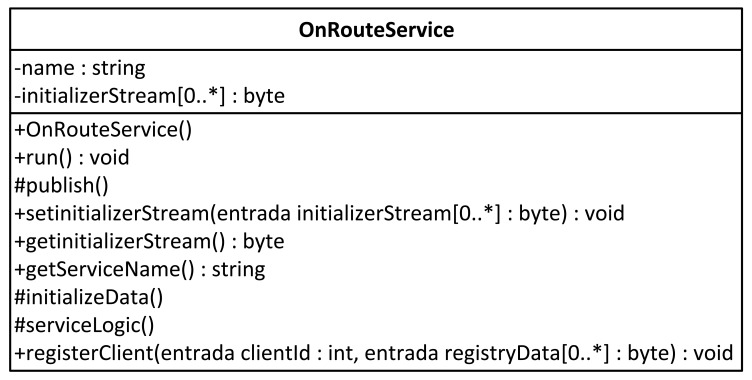
OnRouteService class.

To ensure implementation and to facilitate the work of the programmers, OnRoute encapsulates this flow into an abstract class, known as OnRouteService. Figure 5 shows a series of methods that are run one after another and have to meet two requirements. The first method establishes that the information service has to have a name. Therefore, the class builder demands it. The publication of the service will be automatic and transparent to the user. The second requirement is that all of the services must be able to run continuously. To attain this performance, the abstract class generates instances that are strings. The Thread abstract class is used, providing an abstract method run where we can place the code that runs the string.

The attributes of the OnRouteService class are name, the name of the service, and initializerStream, a stream of data that is passed up to higher layers to construct the interface for the client applications. Each programmer must construct this datum in agreement with what is performed in the remote application.

The operations of OnRouteService include a mandatory constructor. The class is created by invoking the constructor, and it must be given the name of the service. The private publish method is responsible for publishing the service. The programmer must invoke the public setinitializerStream method from the method devoted to initialising variables. The method requires a data stream to construct the interface in the client application. If the service requires initialised data, then the iniatilizeDat operation must be invoked, and this operation has to be implemented by the service developer. In the serviceLogic methods, all of the service logic is implemented. Therefore, its code must be specified by the developer. The run method comes from the Thread abstract class. In this class, the three methods that ensure the run flow of an OnRoute service are run in order. Finally, the logic needed to process the registration of a new client application to the service is specified using the registerClient method. If customer registration is not required, its implementation can be left empty.

#### 4.2.2. Bluetooth Layer (BL)

The Bluetooth layer, or transport layer, is the most complex part of the system on all levels, including the technological, design and implementation levels. This layer is the key part because the functionality provided by any service or system depends on it. Situated just above the CL, this layer is merely a specialisation of a client system's communication layer. The layer has been developed with the greatest level of abstraction possible together with the minimum amount of feedback from the other parts of the system. As a result, any change in this layer has no lateral effect on the rest of the system. Even the replacement of the Bluetooth technology with IEEE 802.11 or any other that may be considered advisable, such as ZigBee, must be totally transparent to the OnRoute services.

The Service Distributor is a BL service that expedites the search for OnRoute services and their connection addresses. This component is implemented as a thread that is continuously listening using a fixed Bluetooth address used by the ISS to direct customers to OnRoute. It is important to remember that the Service Distributor will not store the active services. It will have a list of the services, and it will send them to anybody who logs on. The services will be stored in a data structure created for this purpose. The Service Distributor has to be aware of any changes that occur in this structure. To model this behaviour, we will use design patterns. Specifically, we will use the *Observer* pattern in combination with the *Observable* pattern.

Data are sent over Bluetooth in the form of bytes. Therefore, our container has to be converted into a series of bytes that can be interpreted in a certain way at the other end of the connection. There is an intermediate class, ServiceConnectorParser, responsible for acting as an intermediary with the container. This intermediary returns information in the desired format, thereby uncoupling the container from the Service Distributor. If the container implementation changes, the implementation of the parser is changed, which is transparent to the Service Distributor. Similarly, should the Service Distributor's needs change, the container is not affected.

**Figure 6.**
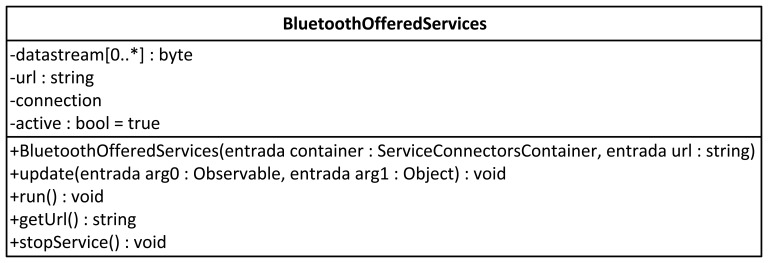
BlueetoothOfferedService.

The Bluetooth services are abstracted by the BluetoothOfferedServices class (see Figure 6). This class has the following attributes: dataStream, a chain of bytes that stores the data to be sent to any customer who logs on to the service, and the url, the service address (Bluetooth) to which the client logs on. This class has the constructor method to which the service storage structure has to be sent, a Bluetooth service url and update, which allows the O*bserver* pattern to operate. This method is called by the observable datum when its status is modified.

The Application Initialiser is another component of the architecture placed in this layer. This component allows applications to obtain their initial data. The initialisation data come from the corresponding service in the SL, *i.e.*, they are defined by the developer. It is OnRoute's mission to carry them from one end of the connection to the other. OnRoute only requires that the data be sent to it in an array of bytes. Therefore, any kind of information can be stored and interpreted at the other end of the connection. The packet that the initialiser sends is built as follows:

[+ Registration address +, + Initialisation data +]

Both data are extracted at the other end in a transparent manner for the developer. The registration address will be used once the developer processes the initialisation data.

Another component of the BL is the Applications Registrar. This component implements a registration system that stores the remote addresses of the customers in case it becomes necessary to send them information or initialise a dialogue. As for the registration system, it must be constantly listening. When an incoming connection is made, it stores a remote address to which it can send data or communicate with the client.

**Figure 7.**
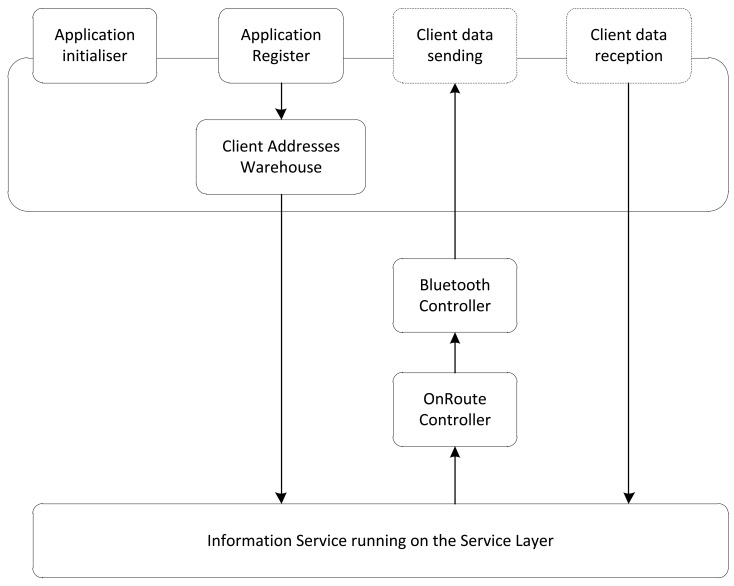
Service Interaction between the Bluetooth layer and the OnRoute services.

With OnRoute, there can be multiple services running at one time. This ability implies that there will be a one-to-one correspondence in the BL. Obviously, there has to be a piece of software that controls everything that happens in the Bluetooth layer and an entry point that allows the control layer to interact. This piece of software is the component of the BL designated as the Bluetooth Controller. Figure 7 shows the interaction between the BL and the OnRoute services.

The Services Container is a fundamental component of this layer. This component stores the services that are going to be activated in the BL and allows them to be managed. We introduced the O*bserver*—O*bservable* design pattern, with the Service Distributor as the observer. Therefore, the Service Container is the observable. The Service Container is supported by a hash structure so that searches are conducted in the shortest possible time. This structure is important when there are many OnRoute services available. The methods of this class are (1) the class constructor, which has no parameters and serves only to construct a hash structure, and (2) the addElement, which serves to obtain a Bluetooth service. Whenever an element is successfully inserted, this change is reported to the Service Distributor, refreshing the list of active services. To delete a service, the class has the deleteElement method, which is sent the name of the service to be deleted. Whenever an element is successfully deleted, this change is reported to the Service Distributor, and the list of active services is refreshed. The getConnector method returns the Bluetooth service that coincides with the name sent. Finally, getData returns all of the information contained in the container in an object.

#### 4.2.3. The Control Layer (CL)

The CL is responsible for coordinating all the information traffic that occurs in the server. Initialising the first service triggers the activation of the OnRoute Controller, which is the name given to the only CL class. The driver has been created using a singleton pattern, which is designed to restrict the creation of objects belonging to a class. The driver's aim is to guarantee that a class only has one instance and to provide a global point of access.

The use of the *singleton* pattern can be a delicate matter in programmes with multiple running threads. If two running threads attempt to create the first instance simultaneously, only one of them should be able to create the object. To solve this issue, we guarantee the mutual exclusion of the constructor by placing a lock on its point of access. In general, this pattern is applied when this class controls access to a single physical resource or when a certain type of data must be available to all of the other objects of the application.

#### 4.2.4. The Infrastructure Layer (IL)

The purpose of this layer is to enable OnRoute to communicate with the transport vehicle systems. This communication makes it possible to obtain data, such as the vehicle identifier, the identifier and the name of the route, stops on the route and position information. Therefore, it is important to develop a layer that allows communication with all systems that provide information of this kind. Unfortunately, the infrastructures used in transport fleet vehicles vary widely. Each implementation of the infrastructure layer will be different with regard to both the services offered and the methods used to access these services. Note that the data that OnRoute demands from the infrastructure are based on Transmodel [13], which is a European specification that describes a data model for public transport systems whose objective is to facilitate the interoperability of transport networks. This layer can be considered to be a state machine. Each state models the vehicle behaviour at some point of its service from the time it is brought into operation until it is taken out of service. The states are marked by a series of data frames that have to be transported by a medium. We will use the User Datagram Protocol (UDP) because it uses notably little bandwidth.

### 4.3. The Client Application

The mobile application is based on the MIDlet specification. The implementation is relatively easy, but it is limited. The operation of the mobile application is simple; we have a MIDlet and a set of screens that we use to browse. The operational diagram consists of selecting an item from the screen. This selection triggers a series of actions. Each of these actions is run on a thread that communicates with OnRoute. The thread obtains data, prints them to the screen, and starts over again. The running of any OnRoute client application is governed by a set of general principles. Specifically, each connection with the ISS or an OnRoute system will be made from a new thread, which is sent a different list such that it will display its data on screens. Before the string is invoked, the list is activated by the setcurrent method. Once the thread prints the elements of interaction with the user on the screen, the user selects one and triggers an event that is handled by the commandAction method, and the whole process begins again. Therefore, the data encapsulated will vary between the different run phases of an OnRoute client application.

To send data, the OnRouteConnectorSender abstract class has been implemented. This class has the connectionUrl stream, and the screen lists and urls as private attributes. The class has the following public operation methods: OnRouteConnectorSender(String connectionUrl, List screen, List urls), run and parse(byte dataToParse[0..*], List screen, List urls). The class is sent the address it has to connect with, and the List object is active on the screen. The object connects and recovers the data. At this moment, the parse method is called by the run method. The parse method is abstract and must be implemented by an OnRoute services developer to enable it to communicate with a service.

For two-way communications between the client application and the service server, a class called CommunicationCenter is provided. This class has the StreamConnectionNotifier type notifier and Boolean type state as private attributes. The public operation methods include stream type CommunicationCenter(String url), haltCenter(), run(), sendData(String url, byte data[0..*]), getUrl() and body(byte data[0..*]. All that is needed is for the user to send the url to which the device is connected. The run method receives the information from OnRoute, and the sendData method allows it to be sent. Finally, every time run receives data, an abstract method, the body, is called to process the data. This abstract method has to be defined by the service programmer.

## 5. Technology

The software produced using OnRoute can be characterised by its capacity to integrate the surrounding physical and technological environments. Consequently, the software can operate autonomously and spontaneously in different environments. We will have a set of OnRoute services with varying availability and a set of OnRoute client applications that will vary in number. If a passenger is in a station that is busy in terms of both passengers and vehicles, the number can be massive. To attain these functionalities, the OnRoute architecture is based on the architecture of pervasive systems [14].

OnRoute uses Bluetooth technology because it is widely supported by users' mobile devices. The information that is exchanged between two Bluetooth units through a set of slots forms a data package, with each device having a unique 48-bit address based on the IEEE 802.11 standard for WLAN. Two or more Bluetooth units that share a single channel from a Bluetooth network are called a piconet. Although the channels have a bandwidth of 1 Mbit, the capacity is reduced to approximately 10 kbit/s as more users join the network. At most, 8 Bluetooth devices can be part of a *piconet*, assuming one of these devices has the role of the master. To minimise the effect of this limitation, the Bluetooth technology incorporates the concept of a *scatternet*, which consists of two or more integrated *piconets*. Currently, the system architecture assumes the use of piconets in the vehicles. Therefore, up to 7 users can access information services that are provided in each vehicle by the system. *Scatternets* are used at stations so that users can access the information provided by the ISS using their mobile devices.

The OnRoute applications that the passengers run on their mobile devices are developed in J2ME. There are two configurations defined in J2ME: the Connected Limited Device Configuration (CLDC) for devices with processing and memory restrictions and the Connected Device Configuration (CDC) for devices with greater resources. CLDC is used for the development of passenger applications.

## 6. A Practical Case: Implementing a Guidance Service

An example of a transport information service is developed in this section, specifically, an on-route guidance service. This service offers a passenger the chance to consult his mobile phone regarding the stops during the journey, subscribe to a destination stop and be advised when the vehicle is about to arrive at that stop. Therefore, the service has to know the name of the route, the stops along the route and the last stop made by the vehicle. All of these data can be obtained from the IL. To begin, we create a new class called InfraestructureService. This class inherits from the OnRouteService abstract class.

First, we define the communications between the service and the IL. This definition is done in the service constructor; we use the PacketTransformer class. This class triggers all of the actions in the IL. We have to send an object from the Connector class, which is responsible for making the network connections with the infrastructure, and the Blackboard class, which is our blackboard object. We then activate the PacketTransformer; therefore, it starts to receive packets from the infrastructure. The packets are processed, and all of the data are initialised. The service is on standby, waiting to receive all of the stops along the route because the mobile application data starter has to be constructed. The source code of this step is as follows:

infrastructure = new PacketTransformer(new Connector(“UDP”, 50000, 1024), blackboard);
infrastructure.start();
while (stopsLinea == null) {
stopsLinea = infrastructure.getStops();
}

In the implementation of the initializeData() method, which is responsible for initialising the data sent to the mobile client application, we recover all of the stops. For each stop, a line of text is generated, which includes the name of the route and its identifier. The source code of this second step is:

data = data + “(” +stop.stop.getName() + “:” !+ stop.stop.getCode() + “)”;

Once the data are generated, it is important that the setinitializerStream(byte[]) method is used to store the data correctly such that it can be used by the higher layers. The source code for this initialisation is

this.setinitializerStream(data.getBytes());

We maintain a hash structure for the registration of client applications. This makes searches as fast as possible. We use a new ExtendedStop structure in the hash that stores the stops along the route. Moreover, this structure will have an array of whole numbers that will make it possible to register the client. The definition of this structure is presented below:

class ExtendedStop {
public Stop stop;
public int clients[];
public int iter = 0;
}

We initialise the data in the initializeData() method, using the fact that we obtained the stop data to generate the initialiser of remote applications. The code of this initialisation is

container.put(stop.stop.getCode(), stop);

The implementation of the registerClient abstract method consists of finding the destination stop in the hash structure, recovering the stop (ExtendedStop structure) and keeping the client identifier in the dedicated array. This is performed as follows:

ExtendedStop stoptarget = container.get(registryData);
stoptarget.clients[stoptarget.iter] = clientId;
stoptarget.iter++;

Before discussing the service logic, we must consider the operation of the infrastructure layer. The base of this layer is the BlackBoard class, where the data received from the infrastructure are written. This structure is an Observable, and our service will be the *Observe*r. Therefore, the update abstract method has to be implemented. Any changes to the data imply a warning to the client because a change means that the stop has changed. The code to get the next stops from the blackboard is

synchronized (this) {
nextstop = blackboard.getnextStop();
newstate = true;
}

The code is executed on a critical section because the variable nextStop controls the logic of the service, and this logic runs on a loop without exit. The loop is iterated only when a change occurs on the blackboard. The next source code illustrates this property:

while (true) {
if (newstate) {
newstate = false;
ExtendedStop nextstop = container
.get(nextstop);
System.out.println(“The next stop is ” + nextstop.stop.getName());
for (int i = 0; i < nextstop.clients.length; i++) {
OnRouteController.getInstance().sendData(this.getName(),nextstop.clients[i], “The next stop is the end of yours trip”.getBytes());
}}}

## 7. Performance

In this section, we describe the tests performed to assess the validity and performance of OnRoute. Because this report presents a framework for the development of ubiquitous information services for the public transport, the tests have been developed to check (1) the quality of the software produced using its development environment and (2) the performance of the services produced.

The system software was developed in accordance with the Test-Driven Development (TDD) model. This methodology for the development and validation of software runs in two stages. In the first stage, unit tests are developed (Test First Development). In the second state, the code is refactored (Refactoring). The methodology allows us to produce clean code that works. In addition, this methodology enables us to assess the flexibility of the software system that is developed because the system has to be flexible enough to allow it to be tested automatically. Otherwise, TDD would not work. The testing was conducted using a test environment that assumes the TDD methodology, the Java Unit Testing Tools (JUnit). The tests were conducted in accordance with the following guidelines.

In the description of the tests performed, we focus on two critical aspects of the system: the Bluetooth communications and the module of utilities used by the programmers who want to develop information services using OnRoute.

For the Bluetooth communications, the first test conducted was the Bluetooth service (BluetoothService). The purpose of this test was to demonstrate the integrity of the data, ensuring that these were consistent. The next test was to verify that the registration of Bluetooth clients was conducted correctly. The class BluetoothServiceRegister is responsible for this action. The purpose of this class is to register the clients who want to use the service. This class should have a Bluetooth address assigned. The test consisted of passing a service address and verifying that the Bluetooth address assigned was correct.

The tests related to the package of utilities were performed with a critical element: the service container. To verify that the services are inserted correctly in the container, two tests were conducted:

1. To verify that the services are inserted correctly, we generated three services and used the getConnector method to verify that they are equal at the object level. In addition, we verified that the names of the services matched.
2. The second test invoked the method responsible for deleting an element and verified whether the element was still in the container.

With these tests, we guarantee that the data are correct in the most critical parts of the system. However, explicit information services are needed to test the system. Three service prototypes have been developed: a route guidance assistant for travellers, a payment system and an inspection system. In general, any service developed with OnRoute must be evaluated with respect to two aspects: the performance of the Bluetooth services and the time required to provide the information requested by the user. The first aspect can be evaluated independent of the information service. The second aspect depends on the logic of the service and the amount of data handled by the service.

To evaluate the performance of the Bluetooth services, the stages through which any Bluetooth communication passes have been analysed. For a client device, the Bluetooth communication processes include searching for the Bluetooth device used by the information server, obtaining the information services provided by the server, establishing a connection to the server, and opening the connection to the server.

For the server, the stages are specifying the attributes of the Bluetooth service offered, creating the connection for the communication with the customer, and opening the connection for the communication with the client.

The performance of the Bluetooth service is heavily conditioned by the searches that the customers' Bluetooth devices perform. As explained above, the OnRoute architecture offers an element belonging to its “run-time” with the mission of facilitating the search, the ISS. The client devices only have to connect to an ISS to obtain information about the Bluetooth servers available. The Bluetooth clients do not need to perform these costly, time-consuming searches. The performance of this configuration has been analysed in two scenarios: a piconet configuration formed by a maximum of 8 Bluetooth devices and a scatternet configuration, where it is possible to have more than 8 devices. The time required for searching ranged between 10 and 20 s, regardless of the configuration.

Tests were also performed to determine the time required to provide information requested by a user from the information service (response time). We conclude that the main factor affecting the response time is the amount of data communicated. Different amounts of data were requested in a piconet configuration. The transmission of data smaller than 512 B was instantaneous (1 s or less), 1 kB was transmitted in approximately 2 s, 79 kB in approximately 15 s and 300 kB required 2 m. An example of an information service that uses small data packages of 512 B or less is the payment system. In contrast, the inspection system requires data packages ranging in size from 512 B to 1,024 B. Finally, considering these results in the implementation of the route guidance assistant for travellers, the maximum size of the packages of data to be transmitted was limited to 1 kB.

## 8. Conclusions and Future Work

OnRoute is a framework for developing and running pervasive software that provides public transport passengers with information services. The OnRoute architecture is deployed in different places on the transport network (vehicles, stations and stops). Moreover, this system has a universal vocation, aiming to reach as many users as possible. OnRoute is flexible enough to be installed in any means of transport.

The information systems currently used in public transport networks handle a large quantity of data that is not always available to the user. OnRoute also serves as a bridge between such systems and the passengers. However, OnRoute does not only cover the needs of passengers; it also covers the needs of those who have to construct information services for passengers, *i.e.*, developers. OnRoute offers any interested developer a simple way to create their services, allowing them to focus on the service's functionality and not on the implementation of complex communication systems between users and machines. OnRoute's architecture is understandable and powerful. Because this system's information services are user-friendly, its use does not require significant resources. OnRoute is a multiplatform development tool; it can be installed in Windows, Mac OS or Linux systems. OnRoute has been tested in Fedora and Ubuntu distributions. This platform is fully documented (using Javadoc) and can easily be integrated into general-purpose development frameworks, such as Eclipse.

There are two main challenges that we must face in the system that we have developed. The first challenge is the limitations of the Bluetooth technology that is used to communicate with users' mobile devices. The second is that the applications are J, such as Android or iOS. Because of these limitations, we propose two future studies. First, we should integrate other technologies to perform local communications with mobile devices, such as IEEE 802.11 and ZigBee. This improvement may be easily accomplished because of the modular and layered architecture of OnRoute. The modules have a great degree of abstraction and a minimum level of coupling among different components. Secondly, we must integrate the Android and iOS technology such that the client applications can be executed on Android and iOS devices. To achieve this goal, we will have to update the OnRoute development framework, but it will not be necessary to change the kernel elements, *i.e.*, the ISS and OnRoute kernel that are executed in vehicles.
